# Association of genetic ancestry with colorectal tumor location in Puerto Rican Latinos

**DOI:** 10.1186/s40246-019-0196-4

**Published:** 2019-02-20

**Authors:** Julyann Pérez-Mayoral, Marievelisse Soto-Salgado, Ebony Shah, Rick Kittles, Mariana C. Stern, Myrta I. Olivera, María Gonzalez-Pons, Segundo Rodriguez-Quilichinni, Marla Torres, Jose S. Reyes, Luis Tous, Nicolas López, Victor Carlo Chevere, Marcia Cruz-Correa

**Affiliations:** 1 Division of Cancer Biology, University of Puerto Rico Comprehensive Cancer Center, PMB 711 Ave. De Diego 89 Ste. 105, San Juan, PR 00927-6346 USA; 2 Department of Biochemistry, University of Puerto Rico Medical Sciences Campus, San Juan, PR USA; 3 Department of Surgery, Division of Urology, University of Arizona College of Medicine, Tucson, AZ USA; 4 Department of Population Sciences, Division of Health Equities, City of Hope Comprehensive Center, Duarte, CA USA; 5 Department of Preventive Medicine and Urology, Norris Comprehensive Cancer Center, Keck School of Medicine of USC, Los Angeles, CA USA; 6 Department of Biochemistry and Medicine, School of Medicine, University of Puerto Rico Medical Sciences Campus, San Juan, PR USA; 7 Colorectal Surgery Clinic, Ashford Presbyterian Hospital, San Juan, PR USA

**Keywords:** Colorectal cancer, Genetic ancestry, Hispanic, Latinos, Colorectal tumors, African ancestry

## Abstract

**Background:**

Colorectal cancer (CRC) is the first cause of cancer deaths among Puerto Ricans. The incidence and mortality of CRC in Puerto Rico continue to be on the rise. The burden of CRC in Puerto Rico is higher than among US Hispanics and is second only to African Americans, thus supporting the importance of studying this CRC health disparity. The genetic background of the Puerto Rican population is a mix of European, African, and Amerindian races, which may account, in part, for the differences observed in the CRC mortality rates among Puerto Ricans. The objective of the study was to assess the role of genetic ancestry in CRC risk and its association with clinicopathological features of CRC tumors in Puerto Ricans.

**Results:**

We used a validated panel of 105 ancestry informative markers (AIMs) to estimate genetic ancestry in 406 Puerto Rican CRC cases and 425 Puerto Rican controls. We examined the association of genetic ancestry with CRC risk and tumor clinicopathological characteristics.

**Conclusions:**

The mean ancestry proportions in the study population were 61% European, 21% African, and 18% Amerindian. No association was observed between genetic ancestry and risk of CRC. However, African ancestry was associated with an increased risk of developing rectal tumors (OR = 1.55, 95% CI 1.04–2.31). Additional studies are needed to fully elucidate the role of African ancestry in CRC carcinogenesis.

## Introduction

Colorectal cancer (CRC) is the third most common cancer diagnosed in the USA [1]. In Puerto Rico (PR), CRC is the leading cause of cancer deaths, accounting for 26.2%, when grouping men and women together [2]. Data from the 2010 US Census determined that Puerto Ricans are the second largest group of Latinos in the USA, which represents approximately 9.0% of the Latino population in the mainland [3]. Furthermore, due to the recent fiscal crisis in the island of PR, it is expected that the number of Puerto Ricans in the US mainland will continue to exponentially increase in the next few years [4].

Compared to non-Hispanic Whites (NHW), Latinos in general have lower CRC incidence rates [5, 6]. However, when comparing CRC incidence rates across Latin American countries, Puerto Ricans have higher CRC incidence rates than Mexicans, Nicaraguans, Hondurans, and most of the other Latin American countries [7]. In the USA, Puerto Ricans living in Florida or California have also been reported to have a higher incidence of CRC than Mexicans living in these states [5, 8]. Moreover, in recent years, CRC incidence and mortality rates among Puerto Ricans have been increasing. CRC mortality rates in Puerto Rico are higher than in US Latinos and are only second to that of US non-Hispanic Blacks (NHB) for reasons that remain unknown [1, 2].

Several studies have shown that the genetic background of the Latino subpopulations varies depending on the region of origin [9, 10]. As an admixed population, Puerto Ricans have a genetic background comprised of varying degrees of three ancestral populations: European, African, and Amerindian (Taínos) [11–13]. Puerto Ricans have higher levels of African genetic ancestry than what has been described in most Latino subpopulations, and a higher contribution of European ancestry when compared to Mexicans [9, 10]. The variations observed in the genetic background across Latino subpopulations reflect differences in the migration patterns of Europeans and Africans into the Americas.

Previous studies have reported associations between Amerindian and African genetic ancestry with an increased risk of a variety of malignancies, including melanoma, prostate, breast, and colorectal neoplasia (adenomas and CRC) [14–18]. Most of the studies examining the association between Amerindian genetic ancestry and cancer have been focused on breast cancer. These studies have shown that US Latinas with higher European ancestry and lower Amerindian ancestry were more likely to develop breast cancer than women with lower European and higher Amerindian ancestry [19, 20]. Moreover, Amerindian ancestry has been associated with worse breast cancer survival [21] and an increased risk of recurrence of acute lymphoblastic leukemia among children [22]. The role of genetic ancestry in CRC risk among Puerto Ricans is unknown. Given the heterogeneous genetic background of this population, differences in genetic admixture may partly explain the differences observed in the presentation and progression of CRC in the Puerto Rican population. In this study, we present the findings of analyses examining the association between genetic ancestry with CRC risk and the clinicopathological features of CRC tumors in the Puerto Rican population.

## Results

### Description of the study population

Cases and controls showed differences in age at recruitment, gender, educational level, family history of CRC, current drinker status, and ever smoked status (Table 1). When compared to controls, individuals with CRC were older (*p* <  0.001), more likely to be male (*p* <  0.001), less educated (*p* <  0.001), and more likely to not have family history of CRC (74.8% had no family history vs. 67.3% in controls; *p* = 0.03) (Table 1). Furthermore, CRC cases were less likely to drink (78.1% were not drinkers; *p* <  0.001) and slightly more likely to have ever smoked (61.9%, *p* = 0.02) than controls (Table 1). The study participants did not significantly differ in marital status (*p* = 0.50), type of health insurance (*p* = 0.07), and BMI (*p* = 0.71) (Table 1).

**Table 1:** Demographics and clinical characteristics by participant status (*n* = 831)

Characteristic	Control (*n* = 425) *n* (%)	CRC (*n* = 406) *n* (%)	*p* value**
Age at recruitment			
< 60 years	293 (68.9)	198 (48.8)	< 0.001
≥ 60 years	132 (31.1)	208 (51.2)	
Gender			
Male	120 (28.2)	211 (52.0)	< 0.001
Female	305 (71.8)	195 (48.0)	
Educational level^a^			
≥ 12 years	155 (61.0)	123 (43.0)	< 0.001
< 12 years	99 (39.0)	163 (57.0)	
Marital status^a^			
Single/divorced/widowed	86 (35.4)	75 (32.5)	0.50
Married	157 (64.6)	156 (67.5)	
Health insurance^a^			
Private	119 (68.8)	184 (65.7)	0.07
Public	54 (31.2)	88 (31.4)	
Other	0 (0.0)	8 (2.9)	
Family history of CRC^a^			
No	226 (67.3)	285 (74.8)	0.03
Yes	110 (32.7)	96 (25.2)	
Current drinker^a^			
No	263 (63.1)	286 (78.1)	< 0.001
Yes	154 (36.9)	80 (21.9)	
Ever smoked 100 cigarettes			
No	290 (69.71)	228 (61.96)	0.02
Yes	126 (30.29)	140 (38.04)	
BMI^a^			
< 20	11 (2.7)	14 (3.6)	0.71
20–24	116 (28.2)	99 (25.1)	
25–29	155 (37.7)	152 (38.6)	
30	129 (31.4)	129 (32.7)	
European ancestry (mean ± SD)	0.610 ± 0.141	0.613 ± 0.122	0.82
African ancestry (mean ± SD)	0.211 ± 0.131	0.207 ± 0.118	0.89
Amerindian ancestry (mean ± SD)	0.180 ± 0.071	0.180 ± 0.071	0.63

^**^p value from chi-square distribution or Fisher’s exact test for categorical variables and Mann-Whitney test for continuous variables

^a^Counts varied due to missing information

### Genetic ancestry and risk of CRC

**Fig. 1:**
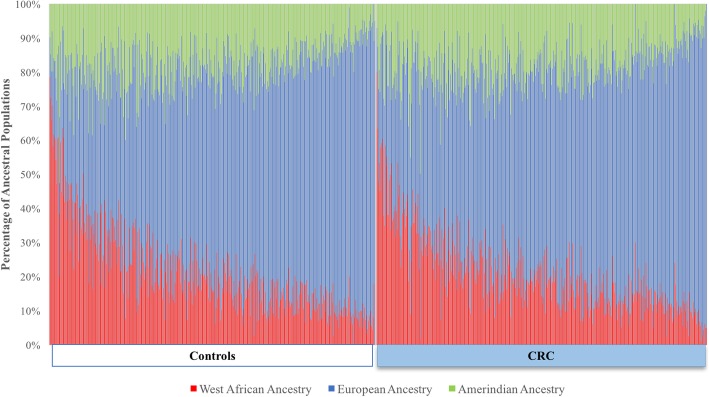
Proportion of ancestral populations by CRC status. Our study populations were divided into controls and CRC cases, and ancestral population proportions were graphed. Each line represents an individual. African ancestry proportions are shown in red, European ancestry proportions are shown in blue, and Amerindian or Taíno ancestry proportions are shown in green

The mean of each ancestral population (European, African, and Amerindian) was calculated according to participant status (Table 1). Controls had 61.0% European, 21.1% African, and 18.0% Amerindian mean ancestral proportions, whereas CRC cases had 61.3% European, 20.7% African, and 18.0% Amerindian (Table 1). No statistically significant differences were observed in the distribution of ancestry proportions between cases and controls. A visual representation of the ancestry proportions by participant status is shown in Fig. 1. As seen in Fig. 1, the proportion of ancestral populations in controls and CRC cases has a similar distribution across the two study groups. When considering genetic ancestry as dichotomous variables, we used a cut-off of the median value of each ancestral population among controls (equal or less than mean vs. more than mean ancestry). However, we did not find evidence of an association between genetic ancestry and CRC risk (Table 2). Genetic ancestry was also analyzed using tertiles and quartiles, but no statistically significant findings were found (data not shown). In addition, genetic ancestry was evaluated using a categorical variable based on quartiles and treated as a continuous variable to determine the variation in risk per 25% increase in the ancestry of each ancestral population, and no statistically significant findings were observed (Table 2). Furthermore, genetic ancestry was evaluated as stratified by age at recruitment (< 60 years and ≥ 60 years) and found no statistically significant changes in the contribution of genetic ancestry by age at recruitment to the development of CRC (Table 3).

**Table 2:** Association of genetic ancestry with CRC status

Ancestry	CRC		
	OR_unadjusted_	OR_adjusted_^b^	OR_adjusted_^c^
Categorical variable^a^			
European ancestry			
≤ (0.63)	1.0	1.0	1.0
> (0.63)	0.95 (0.73–1.25)	0.92 (0.61–1.39)	0.98 (0.64–1.50)
African ancestry			
≤ (0.18)	1.0	1.0	1.0
> (0.18)	1.08 (0.82–1.41)	1.12 (0.74–1.68)	1.16 (0.76–1.76)
Amerindian ancestry			
≤ (0.17)	1.0	1.0	1.0
> (0.17)	1.19 (0.91–1.57)	1.03 (0.68–1.54)	0.99 (0.65–1.50)
Continuous variable^d^			
European ancestry	0.98 (0.87–1.11)	1.01 (0.84–1.22)	1.04 (0.86–1.26)
African ancestry	1.01 (0.89–1.14)	1.01 (0.84–1.21)	1.01 (0.84–1.22)
Amerindian ancestry	1.04 (0.63–1.22)	0.92 (0.77–1.11)	0.89 (0.74–1.08)

^a^Dichotomous variables were analyzed using the median value of each ancestry marker among controls as a cut-off

^b^POR’s adjusted by age at recruitment, gender, education, and family history of CRC

^c^POR’s adjusted by age at recruitment, gender, education, and family history of CRC, ever smoked, and current drinking

^d^Categorical variables were categorized into quartiles and treated as continuous variables to measure the variation in risk per 25% increase of each ancestry marker

**Table 3:** Association of genetic ancestry (categorical variable)^a^ with CRC status by age at recruitment

Ancestry	OR_< 60 years_			OR_≥ 60 years_		
	OR_unadjusted_	OR_adjusted_^b^	OR_adjusted_^c^	OR_unadjusted_	OR_adjusted_^b^	OR_adjusted_^c^
European ancestry						
≤ (0.63)	1.0			1.0		
> (0.63)	0.91 (0.63–1.31)	0.85 (0.49–1.48)	0.94 (0.53–1.67)	0.95 (0.62–1.47)	0.95 (0.51–1.78)	0.97 (0.51–1.86)
African ancestry						
≤ (0.18)	1.0			1.0		
> (0.18)	1.14 (0.79–1.63)	1.11 (0.64–1.92)	1.07 (0.60–1.91)	1.18 (0.76–1.83)	1.25 (0.67–2.35)	1.32 (0.70–2.52)
Amerindian ancestry						
≤ (0.17)	1.0			1.0		
> (0.17)	1.35 (0.94–1.94)	1.39 (0.79–2.43)	1.31 (0.73–2.34)	1.00 (0.65–1.55)	0.76 (0.41–1.40)	0.75 (0.40–1.40)

^a^Dichotomous variables were analyzed using the median value of each ancestry marker among controls as a cut-off

^b^POR’s adjusted by age at recruitment, gender, education, and family history of CRC

^c^POR’s adjusted by age at recruitment, gender, education, and family history of CRC, ever smoked, and current drinking

^d^Categorical variables were categorized into quartiles and treated as continuous variables to measure the variation in risk per 25% increase of each ancestry marker

### Genetic ancestry and CRC tumor characteristics

The pathological and molecular characteristics of CRC tumors was evaluated by each ancestral population and presented in Tables 4 and 5. These tumor characteristics are important to evaluate since CRC subtypes arise through different mechanisms. Furthermore, understanding the contribution of genetic ancestry to these CRC subtypes might increase our knowledge of the biological and molecular pathways that lead to CRC development in Hispanics. The pathological characteristics evaluated in our study included tumor location, tumor stage, and tumor differentiation. As seen in Table 4, individuals with high Amerindian ancestry were more likely to have tumors located in the distal colon (Table 4). However, after adjustments for confounders tumor location was not significantly associated with Amerindian ancestry (Table 5). Moreover, after adjustment for age at recruitment, gender, education, family history, ever smoked status, and current drinking status, individuals with higher levels of African ancestry were three times more likely to present with CRC tumors located in the rectum (OR = 1.55, 95% CI 1.04–2.31) compared to those with low levels of African ancestry (Table 5). No significant associations were found between any of the tumor characteristics, and European or Amerindian ancestry (Tables 4 and 5), even when analyzing genetic ancestry using tertiles and quartiles (data not shown). Additionally, molecular tumor characteristics, such as microsatellite instability, CpG island methylator phenotype (CIMP), and mutations in *KRAS* and *BRAF*, were evaluated in our CRC study population. These molecular markers are associated with different CRC subtypes and are used for the clinical management of CRC [23–28]; thus, it is important to understand if genetic ancestry is associated with these molecular markers. As described in Table 4, *KRAS*-mutated tumors were more frequently found on patients with low Amerindian ancestry (Table 4). However, after adjustments for confounders, none of the ancestral populations were associated with any of the somatic molecular markers studied.

**Table 4:** Distribution of tumor characteristics by ancestral population among patients with CRC

	European ancestry^a^			African ancestry^a^			Amerindian ancestry^a^		
Tumor characteristic	≤ 0.63 *n* (%)	> 0.63 *n* (%)	*p* value^*^	≤ 0.18 *n* (%)	> 0.18 *n* (%)	*p* value^*^	≤ 0.17 *n* (%)	> 0.17 *n* (%)	*p* value^*^
Tumor location (*n* = 336)									
Proximal	40 (22.22)	32 (20.51)	0.53	40 (25.64)	32 (17.78)	0.11	29 (19.85)	43 (23.50)	0.03
Distal	39 (21.67)	42 (26.92)		40 (25.64)	41 (22.78)		29 (18.95)	52 (28.42)	
Rectum	101 (56.11)	82 (52.56)		76 (48.72)	107 (59.44)		95 (62.09)	88 (48.09)	
Tumor stage (*n* = 266)									
0/ I/II	70 (51.09)	59 (45.74)	0.55	60 (46.51)	69 (50.36)	0.74	55 (49.55)	74 (47.74)	0.89
III/IV	53 (38.69)	52 (40.31)		54 (41.86)	51 (37.23)		42 (37.84)	63 (40.65)	
Other/unknown	14 (10.22)	18 (13.95)		15 (11.63)	17 (12.41)		14 (12.61)	18 (11.61)	
Tumor differentiation (*n* = 250)									
High	27 (20.77)	31 (25.83)	0.34	33 (27.05)	25 (19.53)	0.16	24 (22.43)	34 (23.78)	0.80
Low/moderate	103 (79.23)	89 (74.17)		89 (72.95)	103 (80.47)		83 (77.57)	109 (76.22)	
Microsatellite status (*n* = 108)									
Stable	51 (98.08)	52 (92.86)	0.37	51 (92.73)	52 (98.11)	0.36	47 (95.92)	56 (94.92)	0.99
Low/high	1 (1.92)	4 (7.14)		4 (7.27)	1 (1.89)		2 (4.08)	3 (5.08)	
CIMP status (*n* = 83)									
Absent	6 (12.50)	3 (8.57)	0.73	2 (6.06)	7 (14.00)	0.31	6 (13.04)	3 (8.11)	0.73
Low	42 (87.50)	32 (91.43)		31 (93.94)	43 (86.00)		40 (86.96)	34 (91.89)	
KRAS mutation status (*n* = 109)									
Wild type	35 (64.81)	39 (70.91)	0.50	37 (69.81)	37 (66.07)	0.68	31 (57.41)	43 (78.18)	0.02
Mutated	19 (35.19)	16 (29.09)		16 (30.19)	19 (33.93)		23 (42.59)	12 (21.82)	
BRAF mutation status (*n* = 106)									
Wild type	51 (96.23)	47 (88.68)	0.27	49 (96.08)	49 (89.09)	0.27	49 (89.09)	49 (96.08)	0.27
Mutated	2 (3.77)	6 (11.32)		2 (3.92)	6 (10.91)		6 (10.91)	2 (3.92)	

^*^p value from chi-square or Fisher’s exact test

^a^Dichotomous variables were analyzed the median value of each ancestry marker among controls as a cut-off

**Table 5:** Association of tumors characteristics with ancestral markers among patients with CRC

	European ancestry^a^		African ancestry^a^		Amerindian ancestry^a^	
Tumor characteristic^b^	OR unadjusted (95% CI)	OR adjusted^c^ (95% CI)	OR unadjusted (95% CI)	OR adjusted^c^ (95% CI)	OR unadjusted (95% CI)	OR adjusted^c^ (95% CI)
Tumor location						
Proximal	0.84 (0.51–1.39)	0.91 (0.53–1.58)	0.80 (0.48–1.31)	0.83 (0.48–1.45)	1.39 (0.84–2.32)	1.27 (0.73–2.23)
Distal	1.13 (0.70–1.82)	1.13 (0.66–1.91)	1.02 (0.63–1.64)	1.37 (0.80–2.35)	1.69 (1.03–2.75)	1.32 (0.78–2.26)
Rectum	0.85 (0.60–1.21)	0.88 (0.60–1.31)	1.40 (0.98–1.99)	*1.55 (1.05–2.31)*	0.87 (0.62–1.23)	0.75 (0.51–1.11)
Tumor stage						
0/I/II	0.89 (0.60–1.32)	0.88 (0.56–1.38)	1.14 (0.77–1.70)	1.43 (0.91–2.25)	1.28 (0.86–1.90)	1.04 (0.66–1.63)
III/IV	1.03 (0.67–1.58)	1.15 (0.72–1.83)	0.94 (0.61–1.4)	0.93 (0.58–1.48)	1.42 (0.92–2.20)	1.34 (0.83–2.14)
Other/unknown	1.35 (0.66–2.79)	1.53 (0.72–3.23)	1.13 (0.55–2.32)	1.07 (0.51–2.26)	1.22 (0.59–2.52)	1.06 (0.50–2.22)
Tumor differentiation						
High	1.21 (0.70–2.10)	1.25 (0.68–2.29)	0.75 (0.43–1.31)	0.90 (0.49–1.66)	1.35 (0.77–2.35)	1.16 (0.63–2.14)
Moderate/low	0.91 (0.65–1.28)	0.95 (0.64–1.39)	1.15 (0.82–1.62)	1.20 (0.82–1.77)	1.25 (0.88–1.76)	1.11 (0.75–1.63)
Microsatellite status						
Stable	1.08 (0.70–1.66)	1.02 (0.63–1.65)	1.01 (0.66–1.55)	1.23 (0.76–2.00)	1.14 (0.74–1.75)	1.08 (0.67–1.75)
Low/high	4.23 (0.47–38.19)	4.38 (0.48–36.62)	0.25 (0.03–2.23)	0.52 (0.03–2.25)	1.43 (0.24–8.65)	1.39 (0.23–8.46)
CIMP status						
None	0.53 (0.13–2.14)	0.57 (0.14–2.33)	3.47 (0.71–16.88)	3.72 (0.74–18.72)	0.48 (0.12–1.93)	0.42 (0.10–1.74)
Low	0.81 (0.49–1.33)	0.85 (0.49–1.47)	1.37 (0.83–2.26)	1.50 (0.86–2.60)	0.81 (0.49–1.33)	0.83 (0.48–1.44)
KRAS mutation status						
Wild type	1.18 (0.72–1.93)	1.23 (0.72–2.10)	0.99 (0.60–1.62)	1.11 (0.65–1.90)	1.31 (0.80–2.16)	1.20 (0.70–2.07)
Mutated	0.89 (0.45–1.78)	0.86 (0.42–1.75)	1.18 (0.60–2.35)	1.17 (0.57–2.40)	0.49 (0.24–1.02)	0.49 (0.23–1.02)
BRAF mutation status						
Wild type	0.98 (0.63–1.51)	1.01 (0.62–1.63)	0.99 (0.64–1.54)	1.02 (0.63–1.65)	0.94 (0.61–1.47)	0.92 (0.57–1.49)
Mutated	3.17 (0.63–15.91)	2.68 (0.51–14.10)	2.97 (0.59–14.89)	6.36 (0.74–54.37)	0.31 (0.06–1.58)	0.13 (0.02–1.13)

^a^Dichotomous variables were analyzed the median value of each ancestry marker among controls as a cut-off

^b^Controls as reference category

^c^POR’s adjusted by age at recruitment, gender, ever smoked, current drinking, and BMI

## Discussion

Our results showed that differences in the proportion of ancestral populations were not associated with CRC risk in Puerto Rican Latinos. Previous studies have shown associations between differences in the genetic ancestry of an individual with a variety of cancers including breast, melanoma, and prostate [15, 21, 29–34]. To our knowledge, only one other study has examined the association of genetic ancestry with CRC in a Latino population [14]. Hernandez-Suarez et al. studied a population of Colombians and found an increased risk for development of colorectal polyps and CRC per 10% increase in African ancestry [14]. The differences observed between the results for Colombians and our study population could not only be attributed to differences in other risk factors, but also in variations in the percentage of the contribution of the ancestral populations to the genetic background of the population. As shown in the study by Hernandez-Suarez et al., Colombians have lower levels of European (38–44%) and African ancestry (11–13%) than Puerto Ricans (European 60–61%; African 19–21%). Additionally, the percentage of Amerindian ancestry in the Colombian population is much higher (39–45%) than in Puerto Ricans (18%). The variations observed in the percentage of each of these ancestral populations in the genetic background may perhaps contribute to the observed discrepancies on the risk of CRC in each Latino subpopulation. However, variations in the genetic ancestral background may interact with other genetic alterations, environmental exposures, and/or dietary patterns, thereby modulating CRC risk.

Our study also evaluated the association of genetic ancestry with CRC tumor pathological and molecular characteristics. Our results showed that African ancestry increases the odds of having rectal tumors (Table 4). This finding is in contrast with the reported literature that has consistently found an increase in the diagnosis of proximal location of colorectal tumors in African American and Latino patients [35–39]. The localization of colorectal tumors is important since proximal tumors are more likely to be missed during screening colonoscopy [40] and are associated with worse prognosis [38]. In addition, rectal tumors are treated with radiation therapy, which is not generally recommended for proximal tumors [41]. However, comprehensive molecular analyses using data from The Cancer Genome Atlas (TCGA) showed that proximal and distal (rectal) tumors were not significantly different [42]. Therefore, further analyses of the particular genetic pathways present among Puerto Rican and their association with the African ancestry-specific loci are needed to examine the role of African Ancestry and tumor location.

There are several limitations in our study that need to be addressed and considered. First, we did not perform analyses of locus-specific ancestry. This type of analysis could aid in pinpointing the underlying causes of the association of African ancestry with a rectal location in our study population. A second limitation to consider is that not all of the CRC tumors had molecular characteristics data (MSI, CIMP, *KRAS*, *BRAF*) available, and this could potentially affect the statistical power of our analysis to detect differences. Additionally, we did not collect information on annual income for our study participants. It has been shown that socioeconomic status correlates with genetic ancestry [43]. However, we did collect information on educational level, which have been shown they could be used as a proxy for evaluating the effect of socioeconomic status [44–46]. In our study population, the health insurance status was similar across cases and controls. However, educational levels were higher among controls compared to CRC cases. Hence, our final statistical model for the association of ancestry with CRC was adjusted for educational level as well as other relevant socio-demographic factors. CRC cases had a higher number of males, which resembles the population of individuals with CRC in PR where men are more likely to be diagnosed with CRC as compared to women and are diagnosed with CRC > 60 years old [2]. In addition, CRC cases were older than controls, which is comparable to what has been found in other genetic studies [47–50]. Furthermore, adjustment for age in our statistical analyses did not change estimates by more than 15% suggesting that age is not a confounder. Our study controls were shown to have a greater proportion of self-reported family history (non-hereditary) as compared to cases. This might be explained by controls being motivated to seek screening colonoscopies due to cancer history on their families, whereas those that ended up being cases were more likely motivated by symptoms brought on before the diagnosis of CRC [51, 52].

As this is a case-control study, it may occur that some exposures are reported lower in cases than in controls, in part due to changes in habits (due to the development of symptoms) leading up to a diagnosis. Our control group showed a higher consumption of alcohol than our cases. However, the consumption rate was found to be similar to what has been reported in a national study (Puerto Rico Behavioral Risk Factor Screening and Surveillance System) [53]. Puerto Rico Behavioral Risk Factor Surveillance System (BRFSS) is a state-based system of health surveys established in 1984 by the Centers for Disease Control to collect information on health risk behaviors, preventive health practices, and health care access primarily related to chronic disease and injury [53]. Reported exposures on smoking, BMI, and alcohol consumption were similar between the study controls and the PR BRFSS, which supports that our controls are similar to the population of PR. Furthermore, the data collected for alcohol exposure is the current consumption and may reflect changes in the alcohol consumption habits of patients diagnosed with cancer.

We acknowledge that there might be other genetic causes for the development of CRC, not examined in the current investigation. The focus of this study was sporadic (non-hereditary) CRC, thus, we excluded hereditary colorectal cancer cases since their increased risk for CRC development is due to germline mutations in a variety of cancer predisposing genes. Our group has published articles describing the clinical characteristics and mutational spectrum of these hereditary colorectal syndromes in PR [54–56]. Moreover, it is possible that common variants in these cancer predisposing genes increase CRC risk in the PR population. Additional studies to investigate common variants in those genes might be needed to clarify their role in sporadic CRC development.

Our study has several features that strengthen our findings. This is the first report to describe the role of genetic ancestry in the development of CRC in Puerto Rican Latinos, one of the largest groups of Hispanics in mainland US. To the best of our knowledge, this is the first study of CRC in Latinos to include pathological and molecular tumor characteristics in association with genetic ancestry estimations. All of the CRC cases included in the study were pathology-confirmed. Pathology data was obtained from the Puerto Rico Central Cancer Registry (PRCCR) (http://www.salud.gov.pr/Estadisticas-Registros-y-Publicaciones/Pages/Registros/Registro-de-C%C3%A1ncer.aspx) and included all tumor characteristics, such as tumor location, differentiation, and staging. However, the PRCCR does not collect any racial or genetic ancestry data. Thus, this is the first report to show that genetic ancestry was associated with a colorectal tumor characteristic in Latinos and shows the importance of understanding the genetic background in relation to cancer risk. Our results provide additional data on the potential role of African ancestry in specific CRC phenotype and warrant additional studies focused on understanding the mechanisms by which ancestry influences CRC tumor characteristics using techniques, such as admixture mapping. This technique could help pinpoint the genes involved in the observed association and examined additional locus that may increase CRC risk.

## Conclusions

This is the first report to comprehensively examine the association of genetic ancestry with CRC among Puerto Ricans a subpopulation of US Hispanics with a high risk for CRC compared to other Latino subgroups. There was no association between genetic ancestry and risk of CRC development. However, our results showed that African ancestry was associated with rectal tumor location in the Puerto Rican population, which can have an impact on screening and treatment options for patients. Additional studies to elucidate the role of African ancestry in the development of rectal tumors in the Puerto Rican population are needed.

## Materials and methods

### Study population

Participants in this study were recruited through the Puerto Rico Familial Colorectal Cancer Registry (PURIFICAR; http://purificar.rcm.upr.edu), a clinic-based registry of CRC patients in the island that started in 2007. The registry includes individuals with and without cancer with ages ranging from 21 to 85 years old. Cases have pathology-confirmed CRC, and controls are individuals with no previous history of colorectal neoplasia (polyps and/or CRC) who underwent a CRC screening colonoscopy and no polyps and/or cancer were detected. Both cases and controls are recruited into PURIFICAR through gastroenterology and surgery clinics at the University of Puerto Rico Comprehensive Cancer Center, University of Puerto Rico Medical Center, the Isaac Gonzalez Martinez Oncologic Hospital, the Ashford Presbyterian Hospital, and those referred to PURIFICAR by gastroenterologists/surgeons from across the island of PR. Eligibility criteria for this study included being 21 years of age or older, cases having pathology-confirmed CRC, and controls having a negative colonoscopy and no previous history of colorectal neoplasia. Individuals (both cases and controls) with self-reported family history of CRC, not related to a hereditary syndrome, were also included. For this study, family history of CRC was defined as self-reported history of CRC in first-, second-, or third-degree relatives. However, cases with hereditary colorectal cancer syndromes as identified by germline genetic testing were excluded, since the increased risk of CRC on these individuals is due to an inherited mutation in cancer predisposing genes. Our group has previously described the mutational spectrum and clinical characteristics of the hereditary colorectal cancer in PR [54, 55]. Participants diagnosed with colorectal polyps and other non-colorectal cancers were also excluded. Informed consent was obtained for all individuals that participated in the study. The protocol was approved by the University of Puerto Rico Medical Sciences Campus Institutional Review Board.

### Data collection

#### Risk factor and diet questionnaire

All participants completed a questionnaire (in Spanish) modeled by the one used in the Collaborative Family Registries for Colorectal Cancer (Colon CFR) [57]. This questionnaire covers information regarding medical history, reproductive history, diet, physical activity, lifestyle factors, and demographic information. Furthermore, a detailed family history of cancer was obtained for each subject. Certified personnel conducted the informed consent and interviews at the Puerto Rico Consortium for Clinical and Translational Research (https://prctrc.rcm.upr.edu).

#### Pathology and clinical data

Pathology reports were obtained for all cases from the PR Central Cancer Registry (http://www.salud.gov.pr/Estadisticas-Registros-y-Publicaciones/Pages/Registros/Registro-de-C%C3%A1ncer.aspx) or medical records, which were obtained for all study participants. Data on tumor location, tumor stage (TNM stage, tumor differentiation, lymph node metastasis), and number of positive lymph nodes were collected.

### Genomic DNA extraction and quality control

Blood samples were collected from study participants according to standard operating procedures. Lymphocytes were isolated from whole blood using Ficoll density gradient centrifugation, and genomic DNA was extracted from peripheral blood lymphocytes (PBLs) using Qiagen Gentra DNA Puregene Kit (Qiagen) following the protocol provided by the manufacturers. DNA was quantified by spectrophotometry at an absorbance of 260 nm. The purity of DNA was estimated using the 260/280 ratios.

### Colorectal tumor molecular analysis

For CRC cases, microsatellite instability analysis (commercial testing) was obtained from pathology reports when available. Microsatellite instability analysis consists of analyzing a set of genetic markers in normal mucosa and tumor tissue [58, 59]. If the number of repeats differs in tumor tissue and mucosa, then the tumor is classified as having microsatellite instability. *KRAS/BRAF* mutational status (tumor DNA sequencing) was obtained from pathology reports when information was available. For a subset of CRC cases, CpG island methylation phenotype (CIMP) was performed. Briefly, CIMP analysis consists of analyzing the methylation status of the CpG island in the following genes *CAGNA1G*, *CRABP1*, *NEUROG1*, *IGF2*, *RUNX3*, *SOCS1, CDKN2*, and *MLH1* [24, 60, 61] using specific primers*.* DNA is extracted from CRC tumors and bisulfite modified using the methylSEQr Kit (Applied Biosystems) following the manufacturer’s protocol. The bisulfite-modified DNA was used for the subsequent methylation-specific PCR (MSP). Tumors were classified according to CIMP status as follows: CIMP-Zero (0 methylated genes), CIMP-Low (1 to 5 methylated genes), and CIMP-High (6 to 8 methylated genes) [25].

### AIMS panel genotyping

Genomic DNA extracted from PBLs was used to genotype 105 AIMs panel using the Sequenom MassArray iPLEX platform following the manufacturer’s recommendations. The AIMs panel consists of carefully selected SNP markers that were informative for ancestry between three ancestral populations (European, African, and Amerindian) [62, 63]. This AIMs panel was validated for estimating continental ancestry information in a variety of admixed populations, including Puerto Ricans [62, 63]. Genotype SNP calls were generated using the Sequenom TYPER software, which calls allele-specific peaks according to their masses. Genotyping quality control for all SNPs assessed included (1) using blinded duplicate genotyping for 60 DNA samples; (2) a genotype concordance rate of 99% for all markers; and (3) genotyping call rates exceeding 98.5% in all individuals genotyped.

### Inference of ancestry proportions

The STRUCTURE v2.3 software was used to calculate individual ancestry estimates for each participant using a model-based clustering method implemented in the program [64]. Parental population genotypes from Africans, Europeans, and Amerindians were used to determine ancestry estimates under the admixture model as previously described [62, 63], using the Bayesian Markov chain Monte Carlo method (*K* = 3; assumes 3 founding populations) and a burn-in length of 30,000 for 70,000 repetitions.

### Statistical analysis

In this study, we included 1000 subjects recruited into the PURIFICAR, of which 169 did not pass genotyping quality controls, and were excluded. A total of 425 controls and 406 CRC cases, which passed genotyping quality control analysis, were available for this study. Socio-demographic and clinical characteristics were evaluated according to participant status. The socio-demographic variables evaluated in the study included gender, marital status (single/divorced/widowed and married), educational level (< 12 years and ≥ 12 years), type of health insurance (private and public and other), family history of CRC (first- and/or second-degree relatives with CRC and none), current drinker (no and yes), ever smoked 100 cigarettes during their life (no and yes), and BMI (underweight/normal and overweight/obese). In addition, the following clinicopathological variables and tumor characteristics were evaluated: age at cancer diagnosis, tumor location (proximal, distal and rectum), tumor stage (0/I/II, III/IV, and other/unknown), tumor differentiation (high and low/moderate), *KRAS/BRAF* mutation status (wild type and mutated), MSI status (high and stable) and CIMP status (0 and low). Genetic ancestry was modeled both as a continuous variable (% of European, African, and Amerindian ancestry) and as a categorical variable (dichotomous for each ancestral population: less or equal than mean ancestry levels and more than mean ancestry levels), also tertiles and quartiles for each estimated ancestral population (0–25%, 26–50%, 51%–75%, and 76%–100%) were used as categorical variables.

Sociodemographic and clinicopathological characteristics were evaluated according to CRC status using Pearson’s chi-square or Fisher’s exact test, as appropriate for categorical variables, and Mann-Whitney test for continuous variables. Overall, European and African ancestry were highly inversely correlated (*R* = − 0.84), whereas European and Amerindian (*R* = − 0.37), and African and Amerindian (*R* = − 0.18) were less correlated. Therefore, we considered models that mutually adjusted African and European by Amerindian ancestry. Overall, we found that ORs did not differ by more than 5% in models with both ancestral populations; thus, we present models with single ancestral populations. Logistic regression models were fitted to estimate the unadjusted and adjusted odds ratio (OR) and its 95% confidence interval (CI) for the association of CRC status and ancestry, considering the following covariates: age at recruitment, gender, education, family history of CRC, ever smoked, and current drinking. Unadjusted and adjusted ORs did not differ by more than 15% between models for each ancestral population; therefore, we present unadjusted ORs. Among CRC cases, the comparison of tumor characteristics according to the median of ancestry markers among controls was evaluated using Pearson’s chi-square or Fisher’s exact test, as appropriate. Polytomous regression models were fitted to estimate the unadjusted and adjusted odds ratio (OR) and its 95% confidence interval (CI) for the association of ancestry (outcome) and each tumor characteristic (main independent variable), considering the following covariates: age at recruitment, gender, ever smoked, current drinking, and BMI. All statistical analyses were done using STATA 14.0 (STATA Corp., College Station, TX, USA).

## Abbreviations

AIMs: Ancestry informative markers
BMI: Body mass index
CIMP: CpG island methylation phenotype
Colon CFR: Collaborative Family Registries for Colorectal Cancer
CRC: Colorectal cancer
MSI: Microsatellite instability
NHB: Non-Hispanic Blacks
NHW: Non-Hispanic Whites
PBL: Peripheral blood lymphocytes
PR: Puerto Rico
PRCCR: Puerto Rico Central Cancer Registry
PURIFICAR: Puerto Rico Familial Colorectal Cancer Registry
SNP: Single-nucleotide polymorphism
US: United States
